# Supplementary material for: Knowledge, attitude, and practice of Bangladeshi urban slum dwellers towards COVID-19 transmission-prevention: A cross-sectional study
Source: PLOS Glob Public Health. 2022 Sep 6;2(9):e0001017. doi: 10.1371/journal.pgph.0001017 (PMC10021697; doi:10.1371/journal.pgph.0001017)
Supplement: Attachment — Submitted filename: Response to reviewers.docx [file pgph.0001017.s002.docx]

**Response to general editorial comments**

**Comment 1.** Your co-authors have not confirmed authorship of the manuscript. We have resent them the authorship confirmation email; however please check that the above email address for them is correct and follow up personally to ensure they confirm. Please note that we cannot proceed your manuscript until we have received confirmations from all co-authors.

**Response:** Many thanks for this concern. We have communicated with the co-authors for confirming their authorship.

**Comment** 2. Please update the completed 'Competing Interests' statement, including any COIs declared by your co-authors. If you have no competing interests to declare, please state "The authors have declared that no competing interests exist".

**Response**: Many thanks we have now updated the conflict-of-interest statements.

**Comment** 3. Please amend your detailed Financial Disclosure statement. This is published with the article, therefore should be completed in full sentences and contain the exact wording you wish to be published.

ii). State the initials, alongside each funding source, of each author to receive each grant.
iii). State what role the funders took in the study. If the funders had no role in your study, please state: “The funders had no role in study design, data collection and analysis, decision to publish, or preparation of the manuscript.”

**Response:** Many thanks for this comment. We have now updated this statement as follows *“This study was funded by the Swedish International Development Cooperation Agency (Grant No# GR-01455). SSM was the main recipient of the grant. The funder had no role in study design, data collection and analysis, decision to publish, or preparation of the manuscript.”*

**Comment 4**. In the online submission form, you indicated that "The data that support the findings of this study are available from the corresponding author upon reasonable request following the icddr,b’s data sharing policy.". All PLOS journals now require all data underlying the findings described in their manuscript to be freely available to other researchers, either 1. In a public repository, 2. Within the manuscript itself, or 3. Uploaded as supplementary information.

This policy applies to all data except where public deposition would breach compliance with the protocol approved by your research ethics board. If your data cannot be made publicly available for ethical or legal reasons (e.g., public availability would compromise patient privacy), please explain your reasons by return email and your exemption request will be escalated to the editor for approval. Your exemption request will be handled independently and will not hold up the peer review process, but will need to be resolved should your manuscript be accepted for publication. One of the Editorial team will then be in touch if there are any issues.

**Response**: We have now included the deidentified dataset of the manuscript as supporting information. Please see S1 Annex: Deidentified dataset.

**Response to additional editorial comments**

Additional Editor Comments:
The study has important contributions to make because the global Covid-19 pandemic is a contemporary health condition is a public health concern of wide implication in all communities. The review of the manuscript has revealed important flaws in the conceptualization, theoretical and implementation of the problem phenomenon which must be corrected to position the thesis in the study to make the expected contribution to knowledge.

**Comment 1**. The title requires modifications to reflect what the study aimed to accomplish. The variables appearing in the body of the manuscript are not appropriately reflected in the title especially the explanatory variables. The impression is that knowledge and attitude are the only variables to explain the dynamics of Covid-19 transmission prevention in the population, whereas demographic characteristics are also involved. It is very important to determine how these variables were measured in terms of the validity of measures. Title may require some minor modification for clarity to “Knowledge, attitude, and practice of Bangladeshi urban slum-dwellers towards COVID-19 transmission-Prevention: A cross-sectional study”

**Response**: Many thanks we have now revised the title as “Knowledge, attitude, and practice of Bangladeshi urban slum dwellers towards COVID-19 transmission-prevention: A cross-sectional study”

**Comment 2**. The Abstract require some statement modification to establish clarity:
Background: The first COVID-19 case in Bangladesh was detected on 8th March 2020. Since then, efforts are ongoing across the country to raise awareness among the population to prevent the spread of this virus. We aimed to examine the knowledge, attitude, and practice of urban slum dwellers towards COVID-19 and determine to determine which of these factors most significantly predict COVID-19 Transmission-Prevention practice among the population.
The introduction of "factors" is confusing. What are these factors? Challenge to clarity of the aim of the study is the introduction of the word “factor” whereas, the knowledge, Attitude and antecedent factors required to understand the dynamics of the problem phenomenon in the transmission of Covid-19 within the population that require intervention of raising awareness. Which other factors are the authors referring to as “…Multiple regression models were applied to identify the associated factors with KAP scores” in line 30?
The abstract require revision.

**Response**: Many thanks for this comment. We have now revised the abstract. The mentioned line is now revised as follows “We aimed to examine the urban slum dwellers’ knowledge, attitude, and practice (KAP) towards COVID-19 transmission-prevention.” *(Please see abstract)*

We have also removed the ‘factor’ term from the manuscript to avoid confusion. We wanted to see the association of the knowledge attitude and practice scores with the background characteristics of the respondents rather identifying the factors those influencing the dimension of KAP scores.

**Comment 3**. Background to the study:
Line 53 -54 should read; thus, “…such as being in close proximity to an infected person or in an environment where droplets generated from coughs, sneezes or exhalation of an infected person, by touching a contaminated surface among others.”

**Response**: Many thanks, accordingly now we have revised the line. ***(Please page 4, line 67-69)***

**Comment 4**: It is pertinent in proposing what serves as the most effective alternative in the absence of a herd immunity to be conferred by vaccination of large proportion of the population as suggested in lines 54 – 64, to elucidate the theoretical basis for suggesting, in line 63, 64 that knowledge and attitude are significantly associated with the appropriate practice in the prevention and control of spreading COVID-19. This should be grounded in behaviour theories and public health principles of prevention. Unfortunately, this has not been done and very important variable to be considered, perception, has been missed out. The health-seeking paradigm of the of the health belief model (HBM) should have been applied here to validate the constructs used in the development of the instrument. It is noteworthy that public health research has evolved beyond the superficial approaches of the past lacking in grounded theories. The lack of these theories and epidemiological principles to elucidate the likely dynamics of the problem phenomenon to be diagnosed has weakened the study and invalidated any data derived. The paragraph addressing this aspect is central to theoretical and conceptual clarifications that provides the desperately needed validity to the study, in the absence of which invalidates the entire study argument and thesis.

**Response**: Many thanks for this comment. We have now revised the section as follows **“**Therefore, controlling the spread of the virus with transmission prevention related behaviour change intervention is still thought as the most effective measure to protect people from the disease in the absence of pharmaceutical interventions (West et al., 2020). Such interventions can guide how best to promote adherence to individuals’ personal protective behaviours (individual behaviours aimed at protecting oneself and others). However, different enacted personal protective behaviours require different types of interventions guided by various behaviour change principals (Michie et al., 2013). The capability, opportunity, motivation, and behaviour (COM-B) model can be implemented to change the behaviours needed to limit transmission of COVID-19 e.g., cough or sneeze etiquette, using of face masks, keeping physical distance. According to the COM-B model a behaviour-change occurs when capability and opportunity are present and when the person is more motivated to enact that behaviour than any other (Michie et al., 2011). The existence of a behavioural immune system in humans to control epidemic or pandemic is evident in literature (Schaller, 2011). For example, behaviour change during A/H1N1 influenza pandemic in 2009 (Jones and Salathé, 2009; Rubin et al., 2009) and during Zika outbreak (Pinchoff et al., 2019) reduced the transmission of virus. Thus, following the evidence of behaviour theories and public health principles of infectious disease control, several measurement methods have been taken and practices globally to control COVID-19 (Verelst et al., 2016; Yan et al., 2018; Weston et al., 2018; Weston et al., 2020). Globally, countries have adopted various controlling measures to fight against the spread of the virus, notably, frequent and proper handwashing, maintaining social distance, imposing lockdown to limit mobility of people, ensuring isolation or quarantine of the infected and suspected persons (World Health Organization, 2020a; WHO Bangladesh, 2020; DGHS, 2020). Studies showed that the knowledge regarding COVID-19 and measurement precautions and attitude are significantly associated with the appropriate practice, which can play a significant role in the prevention and control of spreading COVID-19 (Ejeh et al., 2020).”

**Comment 5**: The authors are assuming that KAP represents a single construct as stated in the computation of sample size in line 96 “…50% of the adult slum population have proper KAP…”. This would be appropriate if the construct KAP is a weighted-aggregate score of the composite knowledge, Attitude and preventive practice regarding Covid-19 infection.

**Response**: Many thanks for your comment. We have now revised the analysis and updated the knowledge, attitude, and practice score as weighted score in scale of 100. We would like to mention that in this study we have not generated a single composite of KAP score combining knowledge, attitude, and practice scores. We have analysed each of the domain for knowledge, attitude, and practice separately. The KAP has been used as an acronym for knowledge, attitude, and practice and those are separate domains.

**Comments 6.** There is observed serious inconsistency in the statement of course of action to explore the likely predictors of the problem phenomenon. For instance, the study states that “An exploratory study design was used to assess the KAP of urban slum dwellers”, but no where did it state to “…identify the associated factors with KAP scores”. If the study is to identify factors associate with Covid-19 infection prevention, the factors mentioned would include all explanatory variables such as the demographic characteristics, moderating variables derived from the Health Belief Model guiding the design and implementation of the study.

**Response**: Many thanks for pointing out this important issue. We agree with the editor in this aspect. However, we have now revised the manuscript to avoid the confusion and inconsistency. We would like to mention that our aim was to assess the level of knowledge, attitude, and practice among the slum dwellers and how these levels of knowledge, attitude, and practice were associated with the demographic characteristics of the respondents. Now we have revised the terminology accordingly. *(****Please see page 7, line 119-121)***

**Comment 7:** The computed 384 household should individuals per household.
Line 98 should read; “…estimated 384 consenting individuals/households were required to be enrolled for the study”.
Line 99 should read; “…512 individuals/households were selected by multi-stage sampling technique and data collected by telephone interviewer-administered methods with only 476 successful interviews.” Delete line 101.

**Response**: Many thanks. We interviewed one respondent from each household. We have now revised the lines accordingly. ***(Please see page 7,8, lines 132-137)***

Data collection instruments and measures:
It is not enough to mention that “…Instrument was developed by the research team based on published articles related to the assessment of KAP towards COVID-19 and the general guidelines recommended by the Bangladesh government and WHO”, Line 111-112, involves the development and validity of the instrument for data collection and needs to mention, not only published articles related to the assessment of KAP towards COVID-19, but the theoretical foundations of the constructs applied in an attempt to collect data to validate the thesis of factors associated with Covid-19 prevention practices of urban slum dwellers in Bangladesh.
**Response**: Many thanks for pointing out this. We would like to mention that we have not considered the factors affecting the KAP score in this study. We apologies for this confusion arise due to the choice of word. We wanted to assess the level of knowledge, attitude, and practice of slum dwellers towards COVID-19 and their association with background characteristics. We have now revised the manuscript accordingly.

**Comments 8.** For measures and item development, it is customary to have statement of attitude as items instead of questions and likewise with practice. Responses should be in the 4 response Likert-type scales with few examples illustrating actual statements in lines 124 – 130.
The structuring of the variables and measures are weak and flawed as can be seen in the statement of results. It would have been most appropriate to develop weighted aggregate scores and use these to generate results. Attitudes are usually first measured as attitudinal dispositions and subsequently transformed as standardized scores for each participant in the study then frequency distribution into negative and positive attitudes. Practice is usually measured as frequency of practice/behaviour and has response categories of “not at all”, “really”, “occasionally” and always” and not “yes” or “no”. As a result of the flawed measurements (Instrument for data collection, data collected, analysis) the results presented are not able to answer the research questions of what is the level of knowledge of participants in the study, their attitudinal dispositions, their attitudes and level of Covid-19 infection prevention practice?

**Response**: We adopted questions and their corresponding levels from published literature on Knowledge, attitude, and practice towards COVID-19 transmission prevention. We have contextualized the selected questions and pretested them to make sure that the respondents from urban slum can understand all the questions related to KAP. However, we have added a limitation on the lack of taking validation measure during the development of the study instrument in the limitation of this study as *“Thirdly, as the study was a component of a rapid assessment, the questions related to KAP towards COVID-19 transmission control and prevention were adopted and contextualized for Bangladesh from published literature. Health-seeking paradigm of the health belief model could have been applied to validate the study instrument which would strengthen the study findings.”. (****Please see page 25, line 497-501)***

**Comments 9.** By extension, discussion is observed as a re-statement of the results.
**Response**: We have now revised the discussion section. **Please see discussion.**

**Reviewers' Comments to the Author**

Reviewer #1: A) I think it's unfair to say poor knowledge at 48% in conclusion. You are referring to 21% poor as well.

**Response**: Many thanks for this comment. We have now revised the knowledge, attitude, and practice score in three levels: good, moderate, and poor. Accordingly, we have updated the results and revised the conclusion.

B) Were participants incentivized in any form? Please mention.

**Response**: Yes, the respondents were provided BDT 200 (US$2.37) using a mobile financial service compensating for their time spent participating in the study. We have added this line in the manuscript. (**Please see page 9, line 166-167)**

C) 83% were 18-45 and 14% were between 46-60 years. What about remaining 3%. Are they above 60? Please clarify.

**Response**: Many thanks. We have now revised the line as follows “About 82% of the respondents were adults (18–45 years) followed by 14% were aged between 46 to 60 years, and 4% were aged more than 60 years” ***(Please see page 11, line 219-220).***

D) You have mentioned programatic recommendations. Do you have any research oriented recommendations? Would you recommend any other research areas within this context to get better understand the problem?
**Response**: In this study, we used questions/items from published literature. Further studies can be conducted using more rigorously developed and validated KAP instruments. We have added a line in the conclusion. (***Please see page 23, line 465)***

E) I was not able to see the tables in the submission

**Response**: We apologies for this situation. However, we had included all the tables of the manuscript during the submission.

Reviewer #2: Manuscript is provided in a logical and concise fashion.
The only concern is the provision of data in line with the journal's policy. All data should be made fully available during submission of manuscript.

**Response**: Many thanks we have now included the deidentified data as supporting information in the manuscript and also updated the statement on availability of data and materials as “All relevant data contributing to the findings are within the study. The deidentified dataset of this study are available as the supporting information of the manuscript (S1 Annex: Deidentified dataset).”
