# Supplementary material for: Knowledge, attitude, and practice of Bangladeshi urban slum dwellers towards COVID-19 transmission-prevention: A cross-sectional study
Source: PLOS Glob Public Health. 2022 Sep 6;2(9):e0001017. doi: 10.1371/journal.pgph.0001017 (PMC10021697; doi:10.1371/journal.pgph.0001017)
Supplement: Attachment — Submitted filename: Response to reviewers.docx [file pgph.0001017.s003.docx]

**Response to editorial comments**

**Response to editorial comments**

The manuscript presented has improved tremendously, having effected most of the recommended revisions. However, to strengthen the manuscript further, there are observed weaknesses that needs to be address. Most of these are editorial inadequacies but the theoretical issues defining the foundation of the thesis of the study still has flaws.

**Response:** Thank you so much for your comments. We have now revised the manuscript as per the comments.

**Comment 1**. Title modification has been implemented

**Response:** Thank you for this comment.

**Comment 2.** In the abstract: Line 30-31 appear confusing. This should read thus; "A total of 80% or more score for each of KAP variables was classified as ‘good’, scores of less than 80% to 50% as moderate and lower than that as ‘poor’." for clarity. Please revise line 162-163 to align appropriately.

**Response**: Many thanks for this comment and instruction. We have now updated the analysis and accordingly revised the results in the abstract and result section of the manuscript.

Additional Editor Comments (if provided):

Usually, the most appropriate method of categorization that is scientifically-based is the use of quartiles where the first quartile represents poor, the second and third represents below and above average while the fourth quartile represents good level. If you wish to combine the second and the third quartiles to represent average/moderate level, it is equally acceptable. How was attitude determined as good? Usually attitudes are classified as positive or negative, but initially a measure of the attitudinal disposition as a score of the sample in the study is derived and standardized using the Z-score which identifies individuals in the sample distribution with negative attitudes and those with positive attitudes. Therefore, the computations resulting to information expressed in line 35 should be correctly computed.

**Response**: Many thanks for this comment and instructions. Following the instructions, we have performed data analysis and updated the results. We have also added the revised methodology for analysis using quartiles for knowledge and practice and z-score for attitude.

**Background to the Study:**

Most are well expressed but three editorial issues need resolving;

**Comment** 1. Line 84-85 would best be rendered as "Studies showed that knowledge regarding COVID-19 and prevention measures to be taken, and attitudinal dispositions are significantly associated with appropriate infection prevention practice, which has the potential of playing significant role in the prevention and control of community transmission of COVID-19 [20]"

**Response**: Many thanks for this comment and instructions. We have revised the lines with the suggested texts. ***Please see lines 89-92.***

**Comment** 2. Line 98 would better be rendered as, "Evidence from studies showed that…" as three studies have been cited-(30-32).

**Response**: Many thanks for this comment and instructions. We have rendered the line 98 with the suggested texts. ***Please see the line: 107.***

**Comment** 3. Again lines 102-104 would benefit from expressing the significance of the study as; "Therefore, it is important to assess the KAP of slum population towards COVID-19, as a rapid assessment, to evaluate levels of existing knowledge gap and provide basis for policy brief to inform policymakers in the design and implementation of public interventions required in slum areas."

**Response**: Many thanks for this comment and instructions. We have revised the lines with the suggested texts. ***Please see the line:112-115.***

Materials and Methods require minor editorial corrections:

**Comment 1**. Study Design needs to be appropriately expressed. Line 109-111 should be recast to read, thus; "This was a cross-sectional telephone-based exploratory study conducted among 476 adult individuals (>18 years) of 476 households from the selected slums during 31 October to 1 December 2020."

**Response:** Thanks for this comment and instructions. Following the instructions, lines 109-111 have been revised. ***Please see the lines:122-124.***

**Comment 2:** Note line 115 and 144, the acronym for "International Centre for Diarrhoeal Disease Research Bangladesh" should be in upper case letters.

**Response**: Many thanks for this comment. "International Centre for Diarrhoeal Disease Research Bangladesh" use the acronym as icddr,b in their branding, thus we have kept this as icddr,b.

**Comment 3**. The questionnaire development is the major area of concern in the manuscript:
Line 144 -146 failed to indicate the theoretical foundations of the conceptual framework for the study as pointed out in the editor's comments for revision, "Data collection instruments and measures:
It is not enough to mention that “…Instrument was developed by the research team based on published articles related to the assessment of KAP towards COVID-19 and the general guidelines recommended by the Bangladesh government and WHO”, Line 111-112, involves the development and validity of the instrument for data collection and needs to mention, not only published articles related to the assessment of KAP towards COVID-19, but the theoretical foundations of the constructs applied in an attempt to collect data to validate the thesis of factors associated with Covid-19 prevention practices of urban slum dwellers in Bangladesh.

Importantly, the implications of lack of adequate and properly developed theoretical and conceptual clarifications of the constructs in the study has resulted in errors of interpretations leading to inappropriate items developed for especially attitudinal dispositions of the participants in the study. All the 10-item did not represent attitudinal dispositions. In these item, there was observed inability to distinguish what constitutes an attitude and perception, to facilitate appropriate operationalization of attitude in the study. "Attitudes refers to emotional responses expressed emerging from mental dispositions triggered by external circumstances in the environment." Furthermore, the response pattern for attitude and preventive-practice in the instrument are seriously faulty for the constructs. The likert-type response pattern is usually applied depending of the nature of the phenomenon being considered. For example, practice takes into consideration four response-types of "not at all", "occasionally", "often" and "always" Take for instance the items listed for practice of COVID-19 Prevention should not be questions but statements as it appears in the instrument but -("I sneeze into my elbow" with the response of [Not at all]; [Occasionally]; [Often] and [always]" these assess the relative degrees of compliance to preventive requirement and would be scored on the following weighted score of 0, 1, 2, 3"). The second need not have "often" in the statement.

Do you sneeze into your elbow?

Do you often touch your mouth, eye, and nose?

Do you maintain social distance when you go outside?

Do you wear mask when you go outside?

**Response**: Many thanks for this comment. We have now revised the section as follows “The study instrument was developed by the study team in consultation with experts from different fields to check the relevance and make necessary changes according to the study requirement. The development of instrument was grounded on theory of how knowledge and attitudes of individuals influence healthy behavior and motivate them in taking actions towards prevention of infection [35–37].” ***Please see line 158-162.***

**Data Analysis:**

Kindly review line 167 for the acronym of Analysis of Variance and elsewhere this appears such as the tables. This should be One-way ANOVA.

**Response**: Many thanks for this comment. We have now revised it as “One-Way ANOVA” and also throughout the manuscript. ***Please see the lines: 206, also we have changed in Table 5.***

Line 166-169 would better rendered as, "Both descriptive and inferential statistical analysis were performed with the Two tailed t-test for means of variables of two independent samples and the One-way ANOVA for variables of more than two independent samples to test the hypothesis of a statistical difference for each dimension of KAP scores with demographic and socioeconomic categories, separately."

**Response**: Many thanks for this suggestion. We have now revised the lines with the suggested texts. ***Please see the lines: 205-209.***

**Results has minor revision of editorial nature:**

It is never considered appropriate to start the presentation of result with "Table 1 shows…", rather best practice is to begin with the actual result as a reported narrative of important results; very good and poor results, the reader is referred to the tables for other results.

Suggested approach for result presentation for lines 194-197; "The results in the study showed that a total of 476 respondents from 476 households who participated were heads (65%) of households with 82% being adults (18-45 years of age), 18% were older. End the paragraph in line 202 with "(See Table 1)"

Revise line 207 accordingly, and line 255, 268, 296 among others like these.

**Response**: Many thanks for your kind suggestions and comments. Following this comment, we have now updated the presentation of results. ***Please see lines 259, 291, 308, 316, and 350.***

**Discussion:**
Discussion narrative is best started with a brief mention of the objectives of the study to facilitate linking the thesis of the study with the findings and provide coherence of thoughts. Kindly begin line 322 thus; "The study sought to assess the KAP of selected slum dwellers in Dhaka city, Bangladesh towards COVID-19 and found that a small proportion of the sample surveyed demonstrated good knowledge regarding the infection..."

**Response**: Many thanks for your kind instructions and comment. We have now revised the starting points of the discussion section with the suggested texts. ***Please see the lines:381-384***.
